# Massive Sternal Osteophyte Compressing Aortic Arch Branch Graft

**DOI:** 10.1055/s-0041-1736653

**Published:** 2021-12-28

**Authors:** Hesham Ellauzi, Jonathan Cardella, Mohammad A. Zafar, Bulat A. Ziganshin, John A. Elefteriades

**Affiliations:** 1 Department of Surgery, Division of Cardiac Surgery, Aortic Institute, Yale New Haven Hospital, New Haven, Connecticut; 2 Department of Surgery, Division of Vascular Surgery and Endovascular Therapy, Yale New Haven Hospital, New Haven, Connecticut; 3 Section of Vascular Surgery, Yale School of Medicine, New Haven, Connecticut; 4 Department of Cardiovascular and Endovascular Surgery, Kazan State Medical University, Kazan, Russia

**Keywords:** brachiocephalic artery, sternal osteophyte, stenosis

## Abstract

We present a dramatic computed tomography scan demonstrating compression of a brachiocephalic graft by a massive sternal osteophyte, coming to light many years after aortic arch replacement surgery.

A 53-year-old male with Marfan syndrome suffered acute Type A aortic dissection in 1996. He underwent surgical repair with a mechanical aortic composite graft. In 2013, he underwent aortic arch replacement with a side branch to the brachiocephalic artery with a stage-I elephant trunk procedure. In 2014, he underwent carotid-to-carotid bypass surgery followed by a stage-II elephant trunk procedure, with replacement of the descending aorta and side-arm grafting to the left subclavian artery.

During workup in preparation for replacement of an enlarging thoracoabdominal aortic segment, unusual, severe stenosis of the origin of the old brachiocephalic graft was noted on the patient's chest computed tomography (CT) scan. He had no symptoms of cerebral insufficiency. Differential blood pressure readings done in response to the CT findings showed a blood pressure of 150/90 mm Hg on the left and 110/70 mm Hg on the right.

Initially, we could not explain the reason for such narrowing, which was concerning, as the compromised brachiocephalic graft solely supplies blood flow to the brain, encompassing both the right and left carotids (as well as the right vertebral). Upon further review of the CT scan, an astute observer (J.C.) looked beyond the luminal dye shadow, noticing a dramatic posterior sternal osteophyte, which had formed after the previous redo open aortic surgery, compressing the brachiocephalic artery ( Figs. 1 and 2 ; Video 1 ). Although incidents of osteophyte causing native vessel compression were previously reported, 1 2 3 4 we were not aware of a prior such osteophyte-related graft compression.

**Video 1** Thoracic computed tomography scan illustrating brachiocephalic artery compression by a posterior sternal osteophyte that formed following open aortic surgery.


**Fig. 1:**
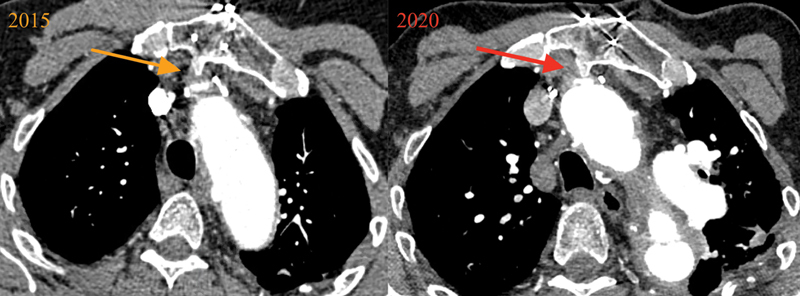
Axial thoracic computed tomography scan images illustrating brachiocephalic artery compression by a sternal osteophyte that formed following open aortic surgery.

**Fig. 2:**
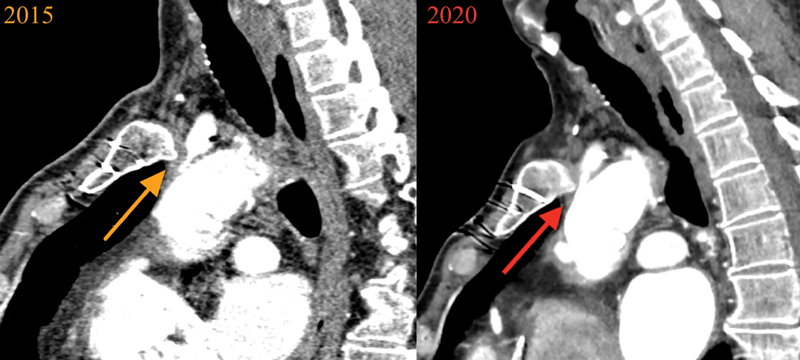
Sagittal thoracic computed tomography scan images illustrating brachiocephalic artery compression by a sternal osteophyte that formed following open aortic surgery.

To augment and secure brain perfusion, a left subclavian to left carotid bypass is planned, so as to avoid a dangerous re-redo sternotomy. This would allow the use of the preexisting carotid-to-carotid bypass to allow bihemispheric cranial flow.
